# Political trust in the handling of the COVID-19 pandemic: a survey in Denmark and Sweden

**DOI:** 10.1186/s44263-023-00009-2

**Published:** 2023-08-09

**Authors:** Thomas Kallemose, Jeanette Wassar Kirk, Elin Karlsson, Ida Seing, Nina Thórný Stefánsdóttir, Karsten Vrangbæk, Ove Andersen, Per Nilsen

**Affiliations:** 1 Department of Clinical Research, Copenhagen University Hospital - Amager and Hvidovre, 2650 Hvidovre, Denmark; 2 Department of Health and Social Context, National Institute of Public Health, University of Southern Denmark, 5230 Odense, Denmark; 3 Department of Health, Medicine and Caring Sciences, Linköping University, 581 83 Linköping, SE Sweden; 4 Department of Behavioral Science and Learning, Linköping University, 581 83 Linköping, SE Sweden; 5 Department of Public Health, University of Copenhagen, 1353 Copenhagen, Denmark; 6 Department of Clinical Medicine, University of Copenhagen, 2200 Copenhagen, Denmark

**Keywords:** Political trust, Pandemic restrictions, COVID-19, Restrictions

## Abstract

**Background:**

The initial responses to the COVID-19 pandemic in Denmark and Sweden differed markedly. Balancing disparate concerns was crucial to generate trust in the COVID-19 restrictions. The aim was to investigate the extent to which there was trust in the handling of the pandemic by the Danish and Swedish governments and public health authorities in each country. A further aim was also to investigate the characteristics of those in Denmark and Sweden who expressed the lowest degree of trust.

**Methods:**

Cross-sectional surveys were conducted in 2021, using web panels that are nationally representative of the socio-demographic characteristics. The population consisted of 2619 individuals from Denmark and 2633 from Sweden, representative of the age, sex and region of residence of the populations aged ≥ 18 years. Trust in government and health authorities was captured in two separate trust questions on a 5-point Likert scale and dichotomized into low trusters and non-low trusters for analysis.

**Results:**

Approximately, 61% of the Danish respondents expressed moderately large or very large trust in the government’s handling of the pandemic. The corresponding proportion for Sweden was 42%. The proportion of low trusters was 11% in Denmark and 34% in Sweden (*p* < 0.001). Moderately large or very large trust in the public health authority’s handling was expressed by 83% of the Danish respondents and 74% of the Swedish respondents. The proportion of low trusters was 5% in Denmark and 17% in Sweden (*p* < 0.001). In both countries, trust was lower among men than among women. Age and education were associated with trust but differed between countries (*p* <  = 0.011).

**Conclusions:**

In this study, differences in trust between Denmark and Sweden and both overall and within socio-demographic factors were observed. However, given the limitations and bias in the study, it is difficult to determine the cause and true size of these differences. With that in mind, we still believe specific populations and subgroups within those populations have the potential to affect trust in handling of the COVID-19 pandemic, and that these should be kept in mind when developing and communicating responses to pandemics.

## Background

The initial responses to the coronavirus (COVID-19) pandemic in two neighbouring Nordic countries, Denmark and Sweden, differed markedly. Denmark rapidly introduced mandatory restrictions to halt the spread of the virus, e.g. shutting all schools, limiting gatherings to 10 people, advising the workforce to stay at home and closing the borders [1, 2]. In contrast, Sweden’s response was characterised by voluntary recommendations and an emphasis on personal responsibility [3, 4]. However, Sweden’s approach changed in response to high rates of COVID-19 cases when the second wave of the pandemic hit in the winter of 2020–2021. This led to a swift development of a Pandemic Law and adoption of many more mandatory restrictions, thus making Sweden’s strategy more similar to Denmark’s [3, 4].

Despite the different responses, Denmark and Sweden are similar in terms of social, cultural and socio-demographic characteristics. Both countries are parliamentary democracies and have public healthcare systems, solid welfare programmes and education that is free of charge [5, 6]. The two countries have a long history of interaction, and the southern part of Sweden belonged to Denmark until 1658. The construction of the Oresund Bridge in 2000 has contributed to creating the Oresund Region as a growing transnational metropolitan area comprising Copenhagen and Malmo [7].

Some studies have investigated the impact of the pandemic responses in the two countries. In a previous study, we showed that there were few differences between the two countries with regard to social distancing categories included in the policy measures, although the policy measures in Denmark consisted of restrictions involving various forms of closure of parts of society to a greater extent [3, 4]. Using three original representative surveys conducted in Sweden and Denmark between March and late June 2020, Nielsen and Lindvall [8] have showed that the Danish population consistently trusted their government and health authorities more than the Swedish population. Swedish trust was politicised and shaped by ideology from the onset of the pandemic, whereas this later became the case in Denmark. Jørgensen et al. [9] have investigated public support for government responses to COVID-19 in eight Western countries, including Denmark and Sweden. Among other findings, they observed the largest degree of support in Denmark and Germany, whereas a moderate level of support was found in Sweden. On a different note, another study showed that differences between Sweden and Denmark’s pandemic strategies did not play a significant role for consumer behaviour [10].

Prior research on public opinion on COVID-19 has primarily focused on three themes. Firstly, it is the rally-around-the-flag effect on political trust and public support for institutions, especially during times of crises [11–16]. The rally-around-the-flag effect describes the tendency of increase in the public’s short-term support for a country’s government or political leaders during periods of crises or war [17]. A study by Esaiasson et al. [18] has shown that trust increased in the early stages of the COVID-19 pandemic in Western Europe. Furthermore, a rally-around-the-flag effect was found in Denmark when it came to trust in the Prime Minister during the lockdown [19]. Secondly, researchers have studied anti-intellectualism understood as a generalised distrust in experts and intellectuals in explaining the public’s engagement with advice from scientists and experts [20–25]. Anti-intellectualism is described as distrust in experts and intellectuals by populists who see them as a class of elites that aim to exploit ordinary people through their positions of power [25]. A former study [26] points out the correlations between ideological polarisation and anti-intellectualism, whereas Merkley et al. [21] have shown that anti-intellectualism matters in its own right, and that it was associated with lower levels of risk perception, social distancing and of COVID-19 concerns. Indicating a link between trust in experts and COVID-19 performance measures. Thirdly, studies have focused on ideological polarisation [27, 28], and some literature argued that ideological polarisation seems to play a role in levels of political trust [8, 27] and for undermining public compliance with health guidelines and expert advice [29].

In both Denmark and Sweden, there was considerable discussion in the public media about whether the measures were sufficient and/or appropriate. Balancing disparate concerns was crucial to generate trust in the restrictions [9]. Trust involves a willingness to be vulnerable and a risk that the other party may not fulfil those expectations [30–34]. Trust may be particularly important in novel and complex situations such as during a pandemic when information is changing constantly [35]. Denmark and Sweden are characterised by high levels of social (or generalised) and political trust [36]. Social trust denotes the trustworthiness of the abstract and generalised other about whom little information exists [37]. Political trust is conceptualised as public support for and confidence in core political institutions, e.g. governments and national public health authorities and often for specific purposes [38, 39]. In opposition to social trust, political trust is more often considered as a political attitude toward an “object”, and in this way, it is conceptualised as more likely to be affected by short-term factors and events [40]. Whereas social and generalised trust is arguably more stable over time, political trust is “all about evaluations of performances” and “particular leaders”, as Eric M. Uslaner argues [39]. Thus, it is responsive to short-term variation in, e.g. the state of the economy and pandemics among other factors. Generally, studies on trust are marked by many perspectives, and how to measure trust has also been disputed. In accordance with other research on trust, we acknowledge that the broad definition of political trust encompasses trust in a specific set of political objects, that is, on the one hand, core institutions of liberal democracy — that is parliament, government, and the justice system as well as the civil service, the police, and the military — and on the other hand, political officeholders, such as party leaders, legislators, and public officials [41]. With regard to the COVID-19 pandemic, and the fact that the two countries initially adopted different responses to the COVID-19 pandemic, we believe that trust in the government and public health authorities is of special interest. It is important to underline that trust in government is likely more driven by partisan and/or ideological considerations [25], while trust in regulatory agencies, such as public health authorities, is more driven by performance. Yet, this should be tempered with the observation that health policy is less dominated by ideological cleavages than many other policy areas in the Nordic region, and that there is broad political support for the public health systems. Socio-demographic factors may also influence political trust; this has been explored both in nonpandemic settings [42] and during the pandemic [43], with mixed results.

Given the importance of trust in authorities and government to achieve compliance with pandemic restrictions, we undertook this study to investigate individuals’ political trust in the handling of the pandemic in Denmark and Sweden. The aim was to investigate the extent to which there was trust in the handling of the pandemic by the Danish and Swedish governments and trust in the handling of the pandemic by the leading public health authorities in each country, the Danish Health Authority in Denmark and the Public Health Agency of Sweden. Additionally,the aim was to investigate the characteristics of those in Denmark and Sweden who expressed the lowest degree of trust. These groups are likely to comply poorly with the restrictions, making it important to understand their characteristics.

## Methods

### Study design and population

Cross-sectional surveys were conducted in Denmark and Sweden in 2021. We used web panels in each country that are administered by Enkätfabriken (Göteborg, Sweden), a company that specialises in survey research. The panels are nationally representative of socio-demographic characteristics. The web panel participants are randomly recruited by telephone and agree to participate in a number of questionnaires each year. They are compensated by means of a system of points that can be exchanged for money or donated to charity.

The population for this study consisted of 5252 individuals: 2619 from Denmark and 2633 from Sweden. The populations were sampled to be representative of the age, sex and region of residence of the Danish and Swedish populations aged ≥ 18 years.

### Data collection

An electronic questionnaire was used to gather the survey data. The questionnaire was distributed via the web panel in April 2021. Responses were kept on Enkätfabriken’s internal servers and were inaccessible to the researchers until all participants had completed the questionnaire.

### Content of the questionnaire

The questionnaire consisted of three questions on socio-demographic characteristics: gender, education and occupation. Information about the respondent’s age was collected by Enkätfabriken. Trust was captured in two questions: trust in how the government and the public health authority in each country had handled the pandemic.

The questionnaire also included questions that were not analysed for this study: a question about the information conveyed by a number of actors, a question about individuals’ altered behaviours in response to the pandemic and questions concerning the acceptability of the COVID-19 pandemic restrictions. The questionnaire also included an open question that asked the respondents to provide suggestions regarding what they believe the governments and public health authorities in Denmark and Sweden could have done differently and/or better to reduce the spread of COVID-19.

### Development of the questionnaire

The questionnaire was developed in Swedish before it was translated into Danish. The Swedish and Danish languages are similar, so there was no need for a back translation process. The Danish and Swedish researchers behind the study discussed any linguistic uncertainties. The Danish translation was adjusted to fit with the Swedish version. All researchers jointly approved the final versions.

A think-aloud study was conducted with the aim of detecting potential problems in participants’ interpretations of various instructions, questions and response items in the questionnaire. The reliability and validity of self-report measures depend on participants interpreting and responding as intended by the researchers. The think-aloud method requires verbalization of thoughts that would normally be silent [44].

The think-aloud study was conducted using an opportunistic sample by recruiting nine individuals from the authors’ social circles, aged 17–71 years, five men and four women, five Danish citizens and four Swedish citizens. The participants were given a paper-and-pen version of the planned questionnaire, with the following written instruction: “We are seeking to find out how the questions in this questionnaire about the COVID-19 pandemic are interpreted. Please fill in the questionnaire and think aloud when doing so. By ‘thinking aloud’ we mean your thoughts, from reading a question until you have decided on a response. Please comment as if you were alone in the room and speaking to yourself”. Four of the authors administered the think-aloud study, each interviewing two or three people. The researcher at each session took notes pertaining to each question of the questionnaire.

The notes from the nine think-aloud sessions were discussed among the authors in a Zoom meeting. This process led to several changes in the various questionnaire instructions, questions and response items. No questions were removed or added. The changes were discussed and agreed upon by all authors at a Zoom meeting.

### Statistical analysis

Demographic variables are presented as frequencies with percentages. Responses on the two trust items were on a 5-point Likert scale (“very high trust”, “high trust”, “neither high nor low trust”, “low trust”, “very low trust”). Those who expressed “very low trust” or “low trust” were labelled “low trusters”.

Proportions of “low trusters” between Denmark and Sweden were compared by chi-squared test. The effects of demographic variables (age, sex, education and occupation) on being a “low truster” within each country were analysed by logistic regression models. Separate models were fitted for each combination low truster variable (government and public health authority) and demographic variable. Models included an interaction term between country and demographic variable, allowing for both within and between country effects. Significance of interactions were tested by Type 3 tests.

Estimates from the logistic regression are presented as odds ratios with 95% confidence intervals and *p*-values. All analyses were performed in R 4.2.1 (R Foundation for Statistical Computing, Vienna, Austria). *p*-values < 0.05 are considered statistically significant.

## Results

Trust in the government’s handling of the pandemic was considerably higher in Denmark than in Sweden (Table 1). Approximately, 61% of the Danish respondents expressed high or very high trust in the government’s handling of the pandemic. The corresponding proportion for the Swedish respondents was 42%. The proportion of low trusters was considerably smaller in Denmark than in Sweden, 11% versus 34% (*p* < 0.001).

**Table 1:** Socio-demographic characteristics of the respondents and expressed trust in the handling of the pandemic by the government and public health authority in Denmark and Sweden

Variables	Denmark, *n* (%)	Sweden, *n* (%)
Age	2619	2633
< 25 years	411 (15.7)	285 (10.8)
25–65 years	1609 (61.4)	1714 (65.1)
> 65 years	599 (22.9)	634 (24.1)
Gender	2619	2633
Women	1394 (53.2)	1448 (55.0)
Men	1225 (46.8)	1185 (45.0)
Education	2601	2628
High school or lower education	784 (30.1)	1102 (41.9)
Vocational education after high school	873 (33.6)	337 (12.8)
University education	944 (36.3)	1189 (45.2)
Occupation	2616	2626
Employed	1354 (51.8)	1577 (60.1)
Student or internship	368 (14.1)	250 (9.5)
Unemployed or long-term sick leave	177 (6.8)	106 (4.0)
Retired	717 (27.4)	693 (26.4)
Trust in the government’s handling of the pandemic	2616	2628
No opinion	21 (0.8)	33 (1.3)
Very low trust	236 (9.0)	441 (16.8)
Low trust	259 (9.9)	446 (17.0)
Neither high nor low trust	512 (19.6)	614 (23.4)
High trust	1027 (39.3)	773 (29.4)
Very high trust	561 (21.4)	321 (12.2)
Trust in the public health authority’s handling of the pandemic	2618	2632
No opinion	20 (0.8)	29 (1.1)
Very low trust	48 (1.8)	192 (7.3)
Low trust	86 (3.3)	263 (10.0)
Neither high nor low trust	295 (11.3)	474 (18.0)
High trust	1269 (48.5)	1067 (50.5)
Very High trust	900 (34.4)	607 (23.1)

The difference was smaller between the two countries with regard to trust in the public health authority’s handling of the pandemic (Table 1). High or very high trust in the public health authority’s handling was expressed by 83% of the Danish respondents and by 74% of the Swedish respondents. Thus, in both countries, trust in the handling of the pandemic by the public health authority was higher than that for the government. The proportion of low trusters was 5% in Denmark compared with 17% in Sweden (*p* < 0.001).

Estimates and *p*-values for characteristic associations and interactions are presented in Table 2. In both Denmark and Sweden, those who were ≤ 65 years were more likely than those aged > 65 years to express low trust in the government’s handling of the pandemic.

**Table 2:** Logistic regression of being a low truster (expressing low or very low trust) with regard to the handling of the pandemic by the government and by the public health authority in Denmark and Sweden

Variable	Comparison	Low trusters in the government’s handling of the pandemic					Low trusters in the public health authority’s handling of the pandemic				
		**Denmark**		**Sweden**		**Interaction**^a^	**Denmark**		**Sweden**		**Interaction**^a^
		**OR (*CI*)**	***p*-value**	**OR (*CI*)**	***p*-value**	***p*-value**	**OR (*CI*)**	***p*-value**	**OR (*CI*)**	***p*-value**	***p*-value**
Age	< 25 years vs. > 65 years	1.52 (1.10–2.10)	0.012	1.48 (1.10–1.98)	0.009	0.872	1.48 (0.75–2.91)	0.258	1.53 (1.07–2.18)	0.018	0.011
	25–65 years vs. > 65 years	1.47 (1.15–1.90)	0.003	1.36 (1.12–1.65)	0.002		2.30 (1.41–3.95)	0.001	1.19 (0.94–1.53)	0.161	
Sex	Men vs. women	2.36 (1.93–2.89)	< 0.001	2.01 (1.70–2.36)	< 0.001	0.223	2.05 (1.44–2.96)	< 0.001	1.61 (1.31–1.97)	< 0.001	0.246
Education	High school or lower education vs. university	0.86 (0.68–1.10)	0.239	1.50 (1.26–1.79)	< 0.001	< 0.001	1.05 (0.70–1.57)	0.822	1.37 (1.10–1.70)	0.005	0.091
	Vocation vs. university	0.88 (0.70–1.12)	0.305	1.32 (1.02–1.70)	0.033		0.68 (0.44–1.05)	0.089	1.25 (0.90–1.71)	0.176	
Occupation	Employed vs. retired	1.51 (1.19–1.94)	< 0.001	1.41 (1.16–1.72)	< 0.001	0.571	1.58 (1.03–2.52)	0.043	1.26 (0.99–1.63)	0.064	0.482
	Student or internship vs. retired	1.53 (1.10–2.11)	0.011	1.47 (1.08–2.00)	0.013		1.47 (0.80–2.64)	0.203	1.67 (1.15–2.41)	0.006	
	Unemployed vs. retired	1.22 (0.78–1.88)	0.367	1.21 (0.51–2.59)	0.644		1.72 (1.12–2.62)	0.013	1.55 (0.91–2.56)	0.094	

*OR* Odds ratio, *CI* Confidence interval. ^a^Type 3 test for country interaction

In Denmark, respondents aged 25–65 years were more than twice as likely as the oldest age group to be low trusters with regard to the Danish public health authority’s handling of the pandemic. In Sweden, those aged < 25 years were more likely to have low trust in the public health authority compared with respondents aged > 65 years. This interaction between country and age was found to be significant (*p* = 0.011).

Men in Denmark were more than twice as likely as women to express low trust in the handling of the pandemic both by the government and by the public health authority. Sweden had a similar pattern; men were twice as likely as women to convey low trust in the government’s handling of the pandemic and 61% more likely than women to have low trust in the public health authority.

Education differed with regard to being a low truster in Sweden but not in Denmark. Compared with university-educated respondents in Sweden, those with high school or lower education and with vocational education were more likely to have low trust in the Swedish government’s handling of the pandemic. Similarly, Swedish respondents with high school or lower education were also more likely than university-educated respondents to express low trust in how the Swedish public health authority handled the pandemic. The interaction between country and education was found to be significant for government trust (*p* < 0.001).

Occupation was a factor with regard to low trusters. Compared with retired respondents in both countries, respondents who were employed and students or those on internships were more likely to express low trust in how the government in each country handled the pandemic. In Sweden, unemployed respondents were also more likely than retired respondents to exhibit low trust in the government’s handling of the pandemic. In Sweden, respondents, who were students or those on internships, had a higher likelihood than retired respondents of reporting low trust in the public health authority’s handling of the pandemic. This was not the case in Denmark, but employed respondents were more likely than retired respondents to express low trust in the public health authority’s pandemic handling.

## Discussion

This study investigated the extent to which there was political trust in the handling of the COVID-19 pandemic in Denmark and Sweden as well as the characteristics of those in each country who expressed the lowest degree of trust in the handling, i.e. the low trusters. Political trust matters for public responses to laws and regulations and to public health authorities’ recommendations [45]. The importance of trust in public authorities for acceptance of and compliance with pandemic restrictions has been established in research on severe acute respiratory syndrome (SARS) and H1N1 (swine flu) influenza [46–48]. It has also been shown that experiencing a crisis can yield changes in public trust in authorities, including governments and researchers [49, 50].

In our study, we observed that the Danish respondents expressed significantly higher trust in their government’s handling of the pandemic than the Swedish respondents, with 23% more low trusters in Sweden. This is consistent with a study by Nielsen and Lindvall [8], who conducted three nationally representative surveys in the two countries between March and June 2020, i.e. approximately a year before our study was carried out (April 2021). They found that Danish respondents consistently expressed higher trust in their government than the Swedish respondents. Similar, but to a lesser extent, Sweden had 12% more low trusters in the public health authority’s handling of the pandemic. As previously described, Denmark and Sweden, as societies, are similar with respect to many issues; however, there are areas where the countries differ which could play a role in explaining the difference observed in the study data. Denmark and Sweden differ markedly in terms of some governance and administrative structures that had implications for the handling of the pandemic. Public authorities in Sweden, including the Public Health Agency, are relatively independent of the government, whereas public authorities in Denmark are led by a minister who is directly responsible for the authority’s activities and has the authority to make many decisions. Ministerial rule is prohibited in Sweden, whereas the minister in Denmark has the power to intervene and steer the everyday work of the authority, although ministers also have to respect the role of the authority as an independent source of expert advice [51]. The Danish government played a crucial and highly active role, using laws and executive orders that allowed Denmark to adopt many mandatory restrictions. In contrast, the Swedish government relied on the Public Health Agency to issue recommendations that encouraged, rather than mandated, people and organisations to behave in certain ways. Sweden did later introduce mandatory restrictions similar to Denmark as a response to the rates of COVID-19 cases. The difference in handling the pandemic in combination with the time of the survey being a few months after the change in response from Sweden could negatively impact the Swedish trust. Since the change in response could be interpreted as admitting that the initial strategy was not working well enough, as well as the COVID-19 rates being an objective measure of this. However, measures a year prior to this survey already showed higher trust in government and health authorities in Denmark compared to Sweden [8]. It is therefore possible that the differences in trust observed in this study are a carryover from the pre-existing difference. However, given that political trust is more susceptible to the short-term effect, it is also possible that the difference in trust during the earlier stages of the pandemic is a consequence of factors specific to that point in time. An example of these factors could be the rally-around-the-flag effect on government trust, which was found to be present in Denmark earlier in the pandemic [19]. Additionally as COVID-19 rates increased in Sweden, trust in the public health authorities would likely fall. Because of this, it is unlikely that the difference between Denmark and Sweden observed here is exsclusively due to the carryover effect.

In both countries, trust in the public health authority’s handling of the pandemic was higher than in the government’s handling. The difference was particularly large in Sweden; almost twice as many respondents expressed high or very high trust in the public health authority’s handling than with the government’s handling. This could be interpreted as high public trust in the Public Health Agency’s expertise and use of voluntary recommendations [52]. These findings are consistent with those of Nielsen and Lindvall [8], who also found that trust in the public health authorities’ ability to guide the country safely through the pandemic was higher than trust in the ability of the government of each country to do so. However, their survey question was slightly different; it asked the respondents to indicate the degree of trust they had in the government and health authority “to guide the country safely through the pandemic” [8]. A contributing factor to this difference in Sweden could be that in contrast to Denmark, politicians and the government in Sweden did not have the main responsibility to inform the public about the status of the pandemic. Instead, the Swedish Public Health Agency was very active, for example they held frequent press conferences on the pandemic to update the public about the latest developments and recommendations.

Several socio-demographic characteristics were observed to be associated with lower trust in the handling of the pandemic by the government and public health authority. In both countries, trust was lower among men than among women. Government trust was lower among those aged < 65 years compared with those ≥ 65 years in both countries; however, differences in the age association were found for trust in the public health authority between the countries. Swedish respondents with a university degree had higher trust in the government than those with lower education. No such pattern was found among the Danish respondents, and the education association was found to be different between the countries. Lower trust in the government was found for employed and student/internship respondents compared to retired in both countries.

The results could suggest some relation between socio-demographic characteristics and trust. As mentioned on the difference in trust in Denmark and Sweden, there could very well be underlying factors confounding these relationships. However, another factor to consider is the way in which pandemic response is communicated. Research on crisis communication, i.e. the collection, processing and dissemination of information to address crisis situations such as a pandemic, has shown the importance of adapting communication for different target groups [53–55]. With regard to the COVID-19 pandemic, the Danish and Swedish governments and public authorities did not use tailored communications based on socio-demographic characteristics [8, 56]. Authorities in the two countries received critique for the lack of adaptation of communication to many immigrant groups beyond translation of messages into multiple languages [57, 58]. Reaching some immigrant groups in both countries proved challenging when the COVID-19 vaccination programmes rolled out, resulting in low vaccination rates among some sub-groups of the immigrant populations in the two countries [59].

This study has some limitations that must be considered when interpreting the results. The study was a cross-sectional survey based on self-reports. Surveys are usually associated with some response bias, i.e. various conditions that can influence responses and make survey data less useful. The panels used by Enkätfabriken (the survey company in charge of the data collection) are nationally representative on socio-demographic variables, but we do not know the respondents’ motivation or interest in responding to the survey about COVID-19. It is possible that those who did respond are the ones most interested in the pandemic and the responses taken in each country to reduce the spread, and thus may not be fully representative of the general population in each country. However, it is difficult to assess how this might have affected the results, i.e. whether they had more or less trust in the authorities’ handling of the pandemic than the broader populations. Trust responses were only measured at a single timepoint; multiple timepoints would have given a more clear picture of the possible change in trust during the pandemic. As described above, this makes interpretation of the observed differences difficult, and we have had to rely on measures from other studies for context. Even though our aim was to investigate associations between specific characteristics, additional measures of political and social attitudes would likely have helped explaining the differences observed. The effect of political ideology on trust may be hard to overcome even during a pandemic. This is also described by Nielsen and Lindvall [8].

## Conclusions

In this study, we observed that low trust toward the government and public health authorities with regard to the handling of the COVID-19 was more prevalent among Swedish responders compared to Danish. Association between socio-demographic characteristics and low trust in government and public health authorities was observed, as well as differences in these associations between Denmark and Sweden. Given the limitations of the study, unmeasured confounders, and lack of repeated measures, we are not able to verify the specific cause for the differences in trust between the countries or the level of bias in the associations. When faced with a pandemic, overcoming pre-existing levels of trust or the effect of political ideology can be difficult. This puts even more weight on factors that can be influenced, such as responses, and how these are developed and communicated, even though this study lacks sufficient evidence to say how specifically this should be done. We would encourage consideration of the specific country and population subgroups within that country when both developing and communicating pandemic responses.

## Data Availability

Pseudonymized version of data may be requested by contacting the corresponding author T. K. (thomas.kallemose@regionh.dk). Approval of the request from the Danish Data Protection Agency will however be needed before transfer of data can be done; the corresponding author will assist with this request.
